# Insufficient Evidence of a Breastmilk Microbiota at Six-Weeks Postpartum: A Pilot Study

**DOI:** 10.3390/nu15030696

**Published:** 2023-01-30

**Authors:** Sophie M. Leech, Morgan C. Gilbert, Vicki L. Clifton, Sailesh Kumar, Kym M. Rae, Danielle Borg, Marloes Dekker Nitert

**Affiliations:** 1 School of Chemistry and Molecular Biosciences, The University of Queensland, Saint Lucia, QLD 4072, Australia; 2 Pregnancy and Development Group, Mater Research Institute, South Brisbane, QLD 4101, Australia; 3 Faculty of Medicine, The University of Queensland, Saint Lucia, QLD 4072, Australia; 4 Centre for Maternal and Fetal Medicine, Mater Mothers’ Hospital, Brisbane, QLD 4101, Australia; 5 Indigenous Health, Mater Research Institute, South Brisbane, QLD 4101, Australia

**Keywords:** microbiome, breastmilk, infant, retrograde inoculation, contamination

## Abstract

Breastmilk is thought to influence the infant gut by supplying prebiotics in the form of human milk oligosaccharides and potentially seeding the gut with breastmilk microbes. However, the presence of a breastmilk microbiota and origins of these microbes are still debated. As a pilot study, we assessed the microbes present in expressed breastmilk at six-weeks postpartum using shotgun metagenomic sequencing in a heterogenous cohort of women who delivered by vaginal (n = 8) and caesarean delivery (n = 8). In addition, we estimated the microbial load of breastmilk at six-weeks post-partum with quantitative PCR targeting the 16S rRNA gene. Breastmilk at six-weeks postpartum had a low microbial mass, comparable with PCR no-template and extraction controls. Microbes identified through metagenomic sequencing were largely consistent with skin and oral microbes, with four samples returning no identifiable bacterial sequences. Our results do not provide convincing evidence for the existence of a breastmilk microbiota at six-weeks postpartum. It is more likely that microbes present in breastmilk are sourced by ejection from the infant’s mouth and from surrounding skin, as well as contamination during sampling and processing.

## 1. Introduction

Breastmilk is the gold standard for infant nutrition with exclusive breastfeeding recommended for the first six months, with continued complementary breastfeeding up to two years and beyond [1]. Breastfeeding has a well-demonstrated influence on the infant gut microbiota [2,3,4], the composition of which is important for future health [5,6,7,8]. Breastmilk is thought to influence the infant gut microbiota in two ways: firstly, by providing human milk oligosaccharides, an indigestible solid component of breastmilk that serves as a prebiotic promoting the abundance of bacteria, such as *Bifidobacterium* [9,10,11,12,13]. Secondly, it has been hypothesised that the breastmilk itself may contain a microbiota that could contribute to the seeding of the infant gut microbiota and hence, influence the health of the infant [14,15,16]. In this study, we use the following definition of a microbiome: it is a ‘characteristic microbial community that occupies a reasonable well-defined habitat and which has distinct physio-chemical properties’, with microbiota referring to the living organisms only, while microbiome also includes ‘their theatre of activity’ [17]. Previous studies have reported the existence of microbes in expressed breastmilk; however, recent reviews of 44 studies respectively highlighted inconsistent findings of the identity of these microbes and their physio-chemical properties [18,19].

Currently, it is unclear from where the microbes in breastmilk originate. Three origin theories exist. In retrograde inoculation, microbes are sourced from the infant’s mouth during feeding; thus, any bacteria contained within the breastmilk were presumably already present in the infant’s digestive tract at some timepoint [20,21]. However, this theory does not explain the presence of microbes in colostrum prior to feeding [22] or in the non-lactating breast tissue [23]; hence, the second theory poses that there likely exists an endogenous microbiota of the breast, which may then be transferred to the milk upon ejection [24]. Finally, it has been proposed that there is an entero-mammary pathway where bacteria translocate from the maternal gut to the breast via immune cells [15,18,25]. However, there currently is no definitive evidence of this occurring in humans.

Previous work characterising the breastmilk microbiota has largely been conducted with 16S rRNA gene amplicon sequencing and has produced mixed results [18]. Generally, the most frequently reported bacteria were *Staphylococcus, Streptococcus, Lactobacillus,* and *Pseudomonas*, which were found in 50% or more studies [18]. Other bacteria, including *Bifidobacterium, Corynebacterium, Enterococcus, Acinetobacter,* and *Rothia,* were reported in one third of studies, with many other genera identified in less than a third of studies [18]. Similarly, a more recent review with more stringent inclusion criteria identified *Staphylococcus, Streptococcus,* and *Lactococcus* as most commonly dominant, and in early milk and colostrum, *Pseudomonas,* some lactobacilli, and bifidobacteria [19]. In addition, some studies fail to identify any bacteria in all or some breastmilk samples [15,26,27]. A large part of the variability in the results may be due to the large heterogeneity in timing of sample collection and the methodology used for sample collection, DNA extraction, sequencing, analysis [18,19], and population demographics [19]. Few studies have attempted to investigate the breastmilk microbiota using shotgun metagenomic sequencing [15,18,27,28,29]. Metagenomic sequencing provides a more detailed and accurate impression of microbiota composition than 16S rRNA gene sequencing, as it sequences all DNA present rather than just one (the 16S rRNA) gene [30]. However, there is a risk with low biomass samples that the larger human genome will dominate the sequence reads and thus, require a greater sequence depth to appropriately capture low abundance species, which comes at a high financial cost [31].

In response to the mixed findings, we aimed to investigate the composition and origin of the breastmilk microbiota by shotgun metagenomic sequencing conducted at two different facilities at six-weeks postpartum in sixteen healthy mother–infant dyads as a pilot study. Furthermore, we aimed to estimate the total bacterial load of breastmilk using standard curves and qPCR.

## 2. Materials and Methods

### 2.1. Sample Collection and Processing

Sixteen breastfeeding women at six-weeks postpartum were selected from the Queensland Family Cohort (QFC) study pilot, an observational, longitudinal birth cohort study [32]. Women were selected based on sample availability, breastfeeding status, singleton pregnancy, gestational age at delivery >36 + 0 weeks, 10th to 90th percentile for birthweight, and maternal pre-pregnancy BMI between 18.5 and 30 kg/m^2^. Women did not have pre-eclampsia, eclampsia, diabetes mellitus, autoimmune disease, or other chronic disease known to potentially influencing the microbiota. Equal numbers of male and female, and vaginally and caesarean delivered infants were selected. All women who delivered by caesarean section had antibiotics administered during labour, one woman who delivered vaginally had antibiotics during delivery, and one woman who delivered vaginally had antibiotics in the previous six weeks for mastitis (Table 1). One woman declared probiotic consumption at enrolment (20 + 5 weeks gestation), and one woman was using probiotics at six weeks post-partum.

QFC breastmilk was collected (up to 10 mL) by the mother by hand expression directly into a sterile container at six-weeks postpartum, stored on ice until aliquoting, and aliquoted for long-term storage at −80 °C prior to DNA extraction. Collection of samples occurred between 6:30 a.m. and 8 p.m.

A large volume of breastmilk (~50 mL) was collected from a single donor (age 35 years, BMI 23.7 kg/m^2^, parity 2) between 6–9 months postpartum for use in method comparison and testing. This was collected by personal pump and frozen immediately at −20 °C. The sample was transported on dry ice and aliquoted for long-term storage at −80 °C.

### 2.2. DNA Extraction from Breastmilk

For sequencing of QFC samples, DNA was extracted from 400 µL of breastmilk per sample using the QIAamp DNA blood kit (Qiagen, Clayton, Australia) with the addition of an initial bead beating step using 0.16 g of mixed zirconia beads in a TissueLyser (Qiagen) for 5 min at 30 Hz. Extraction for sequencing was conducted for all samples at the same time by a single researcher after 1.5–2.2 years of storage at −80 °C. Breastmilk was later extracted using the QIAamp PowerFaecal pro DNA kit (Qiagen) from up to 400 µL of breastmilk in accordance with manufacturer’s protocol for use in qPCR to be comparable with the standard generated by extraction with the same kit as below. Extraction from all samples was again conducted at the same time by a single researcher after 2.9–3.6 years of storage at −80 °C.

For comparison of methods, the single breastmilk sample from 6–9 months postpartum was used. DNA was extracted from 800 µL of breastmilk using the QIAamp DNA mini kit (Qiagen), QIAamp PowerFaecal pro DNA kit (Qiagen), and QIAamp DNA blood kit (Qiagen). The QIAamp DNA mini kit and the QIAamp PowerFaecal pro DNA kit were used in accordance with manufacturer’s protocol, while the QIAamp DNA blood kit was used in accordance with manufacturer’s protocol with the addition of an initial bead beating step with 0.32 g of mixed zirconia beads in a TissueLyser for 5 min at 30 Hz. Whole and ‘skim’ milk were also compared for each of the kits. To create ‘skim’ milk, 10 mL of breastmilk was centrifuged for 20 min at 2683× *g* at −6 °C. The fat layer was then discarded from the top, and the milk was aliquoted into 800 µL amounts. A negative extraction control with Milli-Q water was included for each kit.

### 2.3. Shotgun Metagenomic Sequencing and Analysis

Shotgun metagenomic sequencing was used to assess the composition of the breastmilk microbiota (n = 16). Shotgun metagenomic sequencing with the NovaSeq6000 (Illumina, Singapore) and 2 × 150 bp paired-end chemistry with a target depth of 1 GB was performed as a fee-for-service by two facilities, A and B. Six samples were sequenced by Facility A and ten samples by Facility B. The methods used in the two facilities are described below and compared in Table 2.

In Facility A, library preparation was performed with the Nextera DNA Flex Library Preparation kit (Illumina #20018705) in accordance with manufacturer’s protocol, with reduction of total reaction volume for processing in 96 well plate format. Library prep was run on the Mantis Liquid Handler (Formulatrix, Bedford, MA, USA) and Epmotion (Eppendorf, Macquarie Park, Australia #507500301) automated platform. Quantification and quality control of libraries were performed with Quanti-iT dsDNA HS Assay Kit (Invitrogen, Tullamarine, Australia) and Agilent D1000 HS tapes (Agilent, Mulgrave, Victoria, Australia, #5067-5582) on the TapeStation 4200 (Agilent# G2991AA) per manufacturers’ protocols.

Libraries were pooled at 2 nM per library to create a sequencing pool and quantified in triplicates using the Qubit dsDNA HS assay kit (Invitrogen). Quality control was conducted with the Agilent D1000 HS tapes (#5067-5582) on the TapeStation 4200 (Agilent #G2991AA) as per the manufacturer’s protocol.

In Facility B, library preparation was performed with the Illumina DNA Prep (Illumina #20018705) in accordance with manufacturer’s protocols, with reduction of total reaction volume for processing in 96 well plate format. Library preparation was run on Zephyr NGS (Perkin Elmer, Perth, Australia) automated platform. Quantification and quality control were performed with the Quant-iT ds DNA HS Assay kit (ThermoFisher Scientific, Tullamarine, Australia) and QIAxcel Advanced System (Qiagen, #9002123) using QIAxcel DNA High Resolution Kit (Qiagen, #929002) as per the manufacturer’s protocol.

Libraries were pooled at 2 nM per library to create a sequencing pool and quantified in triplicates using the Qubit dsDNA HS assay kit (Invitrogen) on Qubit Flex Fluorometer. Quality control was performed on the QIAxcel Advanced System (Qiagen, #9002123) using QIAxcel DNA High Resolution Kit (Qiagen, #929002) as per the manufacturer’s protocol.

### 2.4. Metagenomic Sequencing Quality Control and Composition

Post-sequencing, quality control was conducted on the Galaxy platform [33] with tools FastQC Galaxy version 0.73 +galaxy0 [34], ‘MultiQC Galaxy version 1.11 +galaxy0’ [35], ‘Trimmomatic Galaxy version 0.36.6’ with inclusion of an initial Illumina clip step with Nextera (paired-end) sequences [36], ‘Bowtie2 Galaxy version 2.4.2 +galaxy0’ with reference genome, *Homo sapiens* hg38, for removal of host sequences [37], and ‘Samtools view Galaxy version 2.9 +galaxy3’ and ‘Samtools fastx Galaxy version 1.9 +galaxy1’ [38]. All tools were used with default settings unless otherwise specified.

Following quality control, the total number of reads for Facility A per sample was 148,147 ± 62,125 reads with an of average 94.7 ± 2.1% of total reads removed during quality control due to representing human DNA reads. In the sequencing negative control, 10.8% of reads were of human origin prior to removal during quality control, yielding 98,535 reads for analysis. The samples sequenced in Facility B had on average 98.0% (97.33–98.41%) human contamination and yielded 142,296 (73,512–183,647) reads post quality control. No negative sequencing control was available for samples from Facility B as they were reportedly corrupted during the sequencing process although quality control throughout the sequencing preparation indicated no amplification of the sample.

Composition was assessed using MetaPhlAn3 (v3.0.14) with default settings [39].

### 2.5. Comparison of Extraction Kits

Extraction kits were compared by total DNA extracted using Qubit (Thermo Fisher) as well as by amplifying a region of the 16s rRNA gene with primers (Sigma-Aldrich, Macquary Park, NSW, Australia)) (F: 5′-GCAGGCCTAACACATGAAGTC-3′ and R: 5′-CTGCTGCCTCCCGTAGGAGT-3′) by PCR (95 °C for 3 min, then 25 cycles of 30 s at 95 °C, 60 °C, and 72 °C, then 72 °C for 5 min) and gel electrophoresis in a 1% gel to determine whether bacterial DNA was extracted. HyperLadder 50 bp (Meridian Bioscience, Eveleigh, NSW, Australia) was used to determine band size. Later, extraction of total bacterial DNA from each kit was compared with qPCR as below. 

### 2.6. Quantification of Breastmilk Microbial Load

To estimate the number of bacteria present in breastmilk, quantitative PCR with a standard curve was used. Forty cycles of qPCR were conducted with protocol as follows: 50 °C for 2 min, 95 °C for 10 min, 40× 95 °C for 30 s, 60 °C for 30 s, 72 °C for 30 s, then 95 °C for 15 s followed by melt curve from 60 °C to 95 °C.

Bacterial cultures of *Escherichia coli, Staphylococcus aureus,* and a broad-spectrum probiotic mix (Life-space, Melbourne, Australia) containing 15 species of *Bifidobacterium, Lactobacillus,* and *Streptococcus* were cultured (Table 3). *E. coli* and *S. aureus* were cultured individually in Luria Bertani (LB) media overnight at 37 °C, while the probiotic mix was cultured in de Man Rogosa Sharpe (MRS) media (Oxoid, Hampshire, UK) overnight at 37 °C. Bacterial cells were counted using a Helber counting chamber (Hawksley, Sussex, UK), and cultures were combined in equal parts to create mixed cultures of 10^8^, 10^6^, 10^4^, 10^2^, and 10 bacterial cells in either PBS or 800 µL breastmilk. DNA was extracted using the QIAamp Power Faecal Pro DNA kit (Qiagen) per manufacturer’s protocol.

To confirm extraction efficiency, quantitative PCR (qPCR) was performed in triplicate using Rotor-Gene Q (Qiagen) on both mixed cultures in PBS and breastmilk. Each reaction was a total of 10 µL containing 5 µL of QIAGEN QuantiNova SYBR Green PCR mix, 0.4 µL of 10 µM forward and reverse primers, 2.2 µL of H_2_O and 2 µL of DNA. Primers (Sigma-Aldrich) targeting a region of the 16S rRNA gene were used to quantify total bacterial DNA as above. The bacterial load of breastmilk was calculated utilising a line of best fit of the median of technical triplicates of the bacterial standards in Excel v16.67 (Microsoft).

For quantification of bacterial load in QFC breastmilk samples (n = 16), qPCR was performed using the ViiA7 real-time PCR system (Thermo-Fisher), with primers listed as above. Reaction volume was 10 µL as above with the addition of 0.05 µL of ROX per reaction and increase in DNA to 4 µL where possible to compensate for low concentration with corresponding decreases in water. A standard curve was generated by 10-fold serial dilution of DNA extracted from the 10^8^ bacterial cells in PBS. The bacterial load of the breastmilk was then calculated using the standard curve as above.

### 2.7. Statistical Analysis

Figures were generated in GraphPad Prism v9.0.0 and Rstudio v2022.07.0+548 using ‘Phyloseq v1.40.0’ [40] and associated packages ‘Microbiome v1.18.0’ [41], ‘RColorBrewer v1.1-3’ [42], ‘ggpubr v0.5.0’ [43], ‘dplyr v1.0.10’ [44], ‘ggplot2 v3.4.0’ [45], and ‘janitor v2.1.0’ [46]. Statistical tests were determined using GraphPad Prism v9.0.0. Normal distribution was determined with Anderson–Darling test, D’Agostino and Pearson test, Shapiro–Wilk test, and Kolmogorov–Smirnov test. If data set passed all normality tests, unpaired t-tests were used, otherwise Mann–Whitney tests were used. For the categorical variables, Fisher’s exact test was used for comparison. Values are presented as either mean ± standard deviation if they were normal distributed or median (inter-quartile range) if not.

## 3. Results

In the six samples sequenced by Facility A, on average, 94.71 ± 2.15% of reads were of human origin. Five contaminants were identified in the negative control during sequencing, *Porphyromonas gingivalis, Enterococcus faecalis, Streptococcus mutans, Escherichia coli,* and *Acinetobacter baylyi,* which made up on average 82.99% (62.04–87.17%) of the total bacteria detected in each of the breastmilk samples. Of the bacteria that were not present in the negative control, *Staphylococcus epidermidis* was the most commonly present bacteria, appearing in all six samples, followed by *Rhodobacter sphaeroides* in four samples, and *Veillonella atypica* in three samples (Table 4).

In the ten samples sequenced by Facility B, four samples contained no detectable bacteria, five had a single species of bacteria detected consisting of either *Cornyebacterium kroppenstedtii, Cutibacterium acnes* (two samples)*, Staphylococcus epidermidis,* or *Streptococcus mitis.* One breastmilk sample yielded 11 different species; however, for this sample, sequencing occurred at a much greater depth producing 1,030,782 reads post-quality control (~10× the number of reads of the other samples) (Table 5).

### 3.1. Role of the Extraction Method

**Figure 1:**
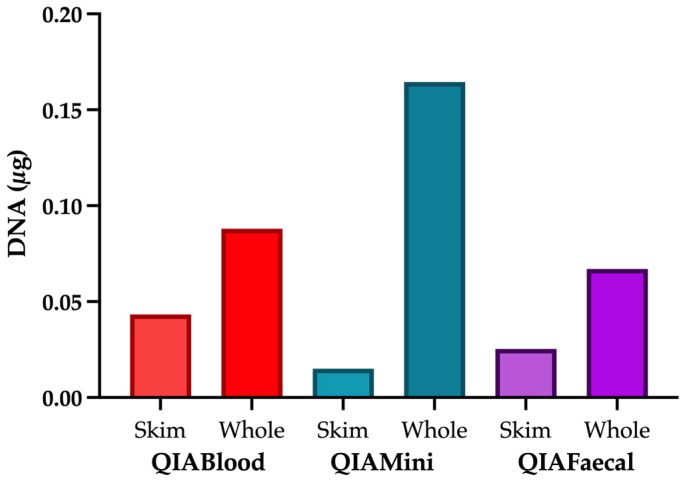
Comparison of total DNA extracted in a single elution of different extraction kits of both whole and skim milk. QIABlood is the QIAamp DNA blood mini kit, QIAMini is the QIAamp DNA mini kit, and QIAFaecal of QIAamp DNA PowerFaecal Pro DNA kit. Extraction controls were all undetectable at less than <0.5 ng/mL.

**Figure 2:**
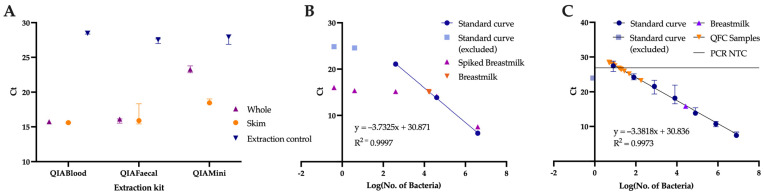
(**A**) Comparison of extraction kits from whole and skim milk. Icon represents median, and error bar represents range of triplicate technical replicates. (**B**) Cycle threshold (C_T_) vs. Log(No. of Bacteria) for testing of extraction efficiency from bacteria in PBS (Standard curve) vs. in breastmilk (Spiked breastmilk). For all but breastmilk (orange inverted triangle) icon represents median of technical triplicates; however, range was too small to display. Median only displayed for test breastmilk sample for ease of viewing. (**C**) Cycle threshold (C_T_) vs. Log(No. of Bacteria) for quantification of bacterial load of QFC breastmilk samples. Icon and error bars represent median and range for standard curve. Orange icons are the median values of the QFC samples (n = 16) with Log(No. of Bacteria) calculated from C_T_ values.

The largest amount of DNA (0.16 µg) was extracted by the QIAamp DNA mini kit (Figure 1); however, as indicated by gel image (Figure S1) and qPCR (Figure 2A), there was a low amount of bacterial DNA extracted by this method, suggesting most of the DNA is of human origin. This is likely due to the absence of a bead-beating step. For this kit, skim milk produced a larger amount of bacterial DNA than whole milk (C_T_ 18.6 ± 0.3 vs. 23.3 ± 0.7). The QIAamp DNA blood mini kit produced similar levels of bacterial DNA to the QIAamp DNA PowerFaecal Pro DNA kit (Figure S1 and Figure 2A). For these kits, there were no differences in the bacterial DNA when comparing whole milk and skim milk (Whole vs. Skim QIABlood: C_T_ 15.7 ± 0.05 vs. 15.6 ± 0.08, QIAFaecal: C_T_ 15.9 ± 0.3 vs. 16.5 ± 1.6). Extraction controls were all below the detection limit (<0.5 ng/mL DNA) but produced C_T_ values of 27.9 ± 0.7 (Figure 1 and Figure 2A).

DNA extraction and amplification from samples is efficient down to 4 × 10^2^ bacterial cells, with a plateau occurring between ~4 and ~0.4 bacterial cells (Figure 2B). Based on the equation generated from 4 × 10^2^, 4 × 10^4^ and 4 × 10^6^ bacterial cells, the breastmilk test sample was calculated to have 5.09 × 10^5^ bacterial cells/mL (Figure 2B). A plateau forms in the spiked breastmilk samples with an added 4 × 10^2^, 4, and 0.4 bacterial cells with C_T_ values similar to the breastmilk sample without spiked bacteria, presumably because the additional spiked bacteria does not significantly increase the total number of bacteria present in the breastmilk sample (Figure 2B). At higher concentrations, breastmilk spiked with 4 × 10^4^ and 4 × 10^6^ bacteria give similar C_T_ values to the bacteria standards (Figure 2B), suggesting little interference in the efficiency of extraction by breastmilk components.

Generating a standard curve of the DNA from 10-fold dilutions from 10^8^ bacterial cells gave a similar bacterial count estimation in the late postpartum breastmilk sample of 4.21 × 10^5^ bacterial cells/mL (Figure 2C). The QFC samples however had 100–1000-fold lower bacterial abundance at 6.51 × 10^2^ (3.37 × 10^2^–2.07 × 10^3^) bacterial cells/mL (Figure 2C), with twelve samples yielding C_T_ values within 1 C_T_ of the PCR NTC or extraction control.

### 3.2. Comparison of Number of Species Detected by Metagenomic Sequencing vs. Bacterial Load

**Figure 3:**
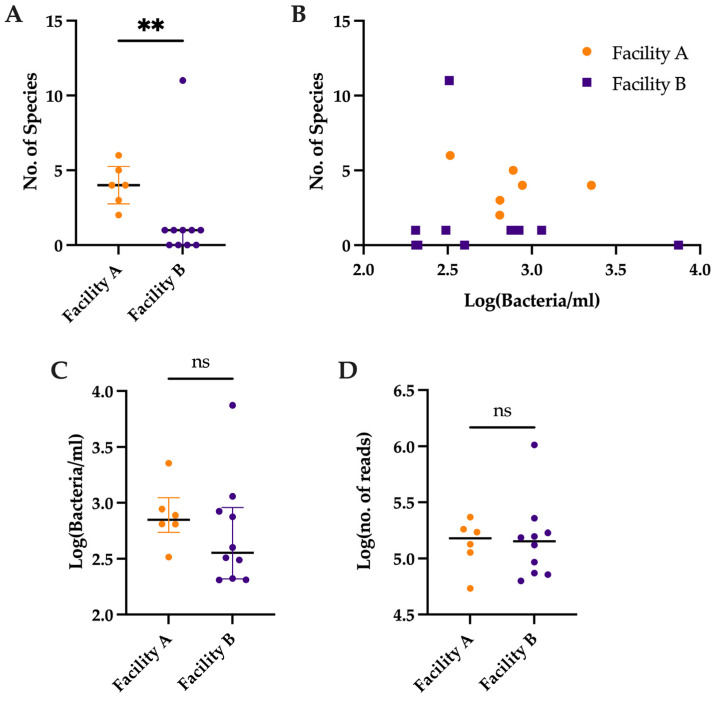
(**A**) Comparison of number of species detected (minus those present in negative control) in sequences from each facility (Median ± IQR). (**B**) Non-significant relationship between Log(Bacteria/mL) and No. of Species detected (minus those present in negative control). (**C**) Comparison of Log(Bacteria/mL) of samples sent to each of Facility A and Facility B. (**D**) Comparison of Log(no. of reads) produced for each sample following quality control. ns *p* > 0.05, ** *p* < 0.01.

Despite the non-significant difference in sequencing depth post-quality control (Facility A: 152,505 (98,626–195,373) reads vs. Facility B: 142,296 (73,512–183,647) reads, *p* = 0.71) (Figure 3D), samples sequenced by Facility A had a significantly greater number of species even after removal of species appearing in the negative control of Facility A (Facility A: 4 (2.75–5.25), Facility B: 1 (0–1), *p* = 0.0046) (Figure 3A). In addition, there was no difference in the number of bacteria estimated by qPCR in the samples sent to each facility (Facility A: 710 (565–1223) bacteria/mL vs. Facility B: 361 (209–916) bacteria/mL, *p* = 0.26) (Figure 3C) or any significant relationship between the number of bacteria estimated by qPCR and the number of species detected in either group (Facility A: R^2^ = 0.07, *p* = 0.61 vs. Facility B: R^2^ = 0.03, *p* = 0.65) (Figure 3B).

## 4. Discussion

Our results do not provide strong evidence that breastmilk has a true microbiota. There is little difference in the amount of total bacterial DNA extracted from whole vs. skim breastmilk supporting the 16S rRNA gene amplicon sequencing results reported [47]. While the overall yield in DNA was similar between kits, it is likely the bead-beating steps included with the QIABlood and QIAFaecal kits increased the amount of bacterial DNA extracted as bead-beating aids in the destruction of the cell wall of Gram-positive bacteria [48].

Breastmilk constituents do not affect qPCR efficiency as the results from spiked bacterial DNA in breastmilk were very similar to those of spiked bacterial DNA in PBS. By qPCR, the bacterial abundance in breastmilk samples at six-weeks postpartum was similar to negative extraction and water controls, at the lower limit of the standard curve where a median of 12.5 bacterial cells was estimated to be present in each reaction. This equates to 6.45 × 10^2^ (3.12 × 10^2^–8.69 × 10^2^) bacterial cells per mL breastmilk, indicating a very low bacterial biomass highly susceptible to contamination. This finding is similar to bacterial abundance measured from plate cultures conducted previously of <10^3^ CFU/mL [49] and 1.73–4.36 × 10^2^ CFU/mL [28]. For comparison, high abundance bacterial body sites, such as skin, may contain up to 10^7^ bacteria/cm^2^ and stool up to 10^11^ bacteria/gram [50]. In contrast, the placenta in which the presence of a microbiota is strongly contested, produces a similar bacterial count of ~10^2^ bacteria with qPCR [51] or values similar to negative controls [52,53], which reflects what is seen in the six-week postpartum breastmilk samples in this study.

The low microbial biomass of breastmilk aligns with what is known about the physiology of lactation and breastmilk. During the first few months, exclusively breastfed infants feed 8–12 times a day or roughly every 2 to 3 h [54]. With each feed, they remove the majority of stored milk, with milk being produced continuously [55]. In addition, and perhaps more importantly, breastmilk is known to contain multiple anti-microbial compounds, including lactoferrin, lysozyme, immunoglobulins, and other immune cells [21,56]. Thus, it is logical there is minimal bacterial accumulation in breastmilk. As the bacterial abundance in the six-weeks postpartum breastmilk samples were comparable to the extraction and water controls, and the extraction controls were not sequenced, the species abundance results from the metagenomic sequencing should be interpreted with caution.

Our metagenomic sequencing revealed the majority of bacterial DNA present in our expressed breastmilk samples from six-weeks postpartum were bacteria that are routinely present on skin and in the oral cavity, similar to what has been reported previously [14,15,18,57]. The detected bacteria could reflect bacteria present on the skin and ducts of the surrounding breast and present in the infant’s mouth during feeding. This is in line with previous work which found treatment with PMA (propidium monoazide) prior to extraction of DNA from fresh breastmilk, to distinguish viable from non-viable cells, resulted in reductions in abundance of oral bacteria, such as *Rothia mucilaginosa, Streptococcus salivarius,* and *Streptococcus mitis*, but increases in abundance of the skin bacterium, *Cutibacterium acnes* [21]. These results indicate oral microbes detected in breastmilk are likely deposited during feeding but then struggle to survive once outside their environmental niche and hence, do not represent a persistent population of bacteria in breastmilk [21]. Our results thus further support the theory that the majority of detectable bacteria are externally sourced from the infants mouth and surrounding skin, rather than from an internal pathway, such as entero-mammary translocation [25].

*Bifidobacterium* was detected at low abundance in a single breastmilk sample that was sequenced at a greater than requested depth by Facility B. Previously, studies interpreted findings of *Bifidobacterium* in breastmilk samples as suggestive of the existence of an entero-mammary pathway [15] as *Bifidobacterium* is not a typical resident of the mouth or the skin [18]. However, to counter this, all bacteria present in the gut must have at some point passed through the mouth as it is the entry to the digestive tract and therefore, may be passed to the breast during feeding. Given that we know that samples were sequenced alongside infant stool samples which have a high abundance of *Bifidobacterium,* and we lack a negative control for samples from Facility B, cross-contamination is a possibility we cannot rule out.

The low biomass nature of expressed breastmilk brings an array of issues in studying its contents. Low biomass samples are highly susceptible to contamination, which is practically unavoidable in the process of sampling and processing, even with rigorous laboratory techniques [58,59]. The majority of bacteria identified in sequenced samples from Facility A were also found in the negative sequencing control. Interpretation of negative controls can be difficult as bacteria identified in the negative control are not necessarily representative of contamination sourced from the environment, kit reagents, or person processing the samples but may be sourced from either cross-contamination or cross-indexing from samples [60]. In our samples however, we are inclined to hypothesise the bacteria found in the sequencing negative control are due to external contamination of the samples as they were not identified in any of the samples from Facility B, which is unusual given their consistently high abundance. Additionally, the identified bacteria, particularly *Acinetobacter* [51,58,59], *Escherichia* [58], *Enterococcus* [51], and *Streptococcus* [58], have all previously been identified as contaminants. These genera have also been reported as members of the breastmilk microbiota in a recent review [18], though it is unclear if negative control samples were sequenced at the same time in each of the individual studies.

In this study we used metagenomic sequencing to characterise the microbes of expressed breastmilk. Metagenomic sequencing’s advantage over 16S rRNA gene amplicon sequencing is that it sequences all DNA present rather than only the 16S rRNA gene [30]. This allows for a more accurate and detailed picture of the microbiota; however, it may flood the results with largely human reads in low biomass samples as the human genome is far larger than the bacterial genome, and contamination with human cells is common in these low biomass samples [31]. This could result in lower abundance species being missed [31]. However, the results presented here from Facility A and the single highly sequenced sample from Facility B are comparable with many studies conducted by 16S rRNA gene amplicon sequencing [14,15], suggesting the large number of human reads has not significantly impacted the profile. It is important to note some studies have also observed an absence of bacterial sequences from some breastmilk samples using 16S rRNA gene amplicon sequencing [15,26]. This suggests all techniques used for assessing the presence of bacteria, e.g., metagenomic sequencing, 16S rRNA gene amplicon sequencing, and in vitro culturing of breastmilk samples, have shown similar results indicating a very low number of bacteria, which may reflect contamination with skin and oral bacteria, rather than a true breastmilk microbiota.

Given the low biomass of breastmilk, the absence of physiological conditions allowing for significant bacterial growth, and expressed breastmilk consisting largely of a low number of skin and oral bacteria, it is likely the microbes detected in expressed breastmilk are largely added during the ejection process. Therefore, they likely represent a combination of skin microbes that have migrated into the mammary gland along with transient oral bacteria rather than a resident breastmilk microbiota at six-weeks postpartum. Thus, in line with the definition of microbiome as a ‘characteristic microbial community occupying a reasonable well-defined habitat which has distinct physio-chemical properties’, with microbiota referring to the living organisms only, while microbiome also includes ‘their theatre of activity’ [17], our results do not provide evidence of a breastmilk microbiota at six-weeks postpartum.

There remains a possibility that gut bacterial species exist in breastmilk at levels below the detection limit that may colonise the infant gut. However, gut bacteria are also transmitted between adult individuals through other pathways [61], which could contribute significantly to the development of the infant gut microbiota development, and should be equally considered when contemplating the origins of the infant gut. In addition, it is possible the high antibiotic use in this cohort, with 10 out of 16 samples affected by antibiotics in the previous six weeks, may have reduced the bacterial load of these samples. However, for 9/10 samples these antibiotics were administered six weeks prior during labour, and there was no significant difference in the bacterial count between samples that has been exposed to antibiotics and those that had not (Figure S2).

## 5. Conclusions

The results reported here in this pilot study do not provide strong evidence for the existence of a breastmilk microbiota. Rather, the evidence presented here aligns more closely with the source for expressed breastmilk microbes being the duct upon milk ejection, which is likely populated with skin microbes along with oral microbes sourced from the infant mouth during feeding. Further research quantifying breastmilk microbial count in a larger cohort is needed to confirm these findings as well as metagenomic sequencing with more sampling and environmental controls to further identify the origin of these microbes.

## Figures and Tables

**Table 1:** Participant characteristics of samples sent to Facility A and B for metagenomic shotgun sequencing. * Incomplete parity data: n = 4 Facility A, n = 7 Facility B.

	Facility A (n = 6)	Facility B (n = 10)	*p*-Value (Facility A vs. Facility B)
Maternal age (years)	30.67 ± 5.24	32.10 ± 3.81	0.54
Maternal ethnicity			
Caucasian	4	9	0.52
North-East Asian	2	0	0.13
Southern and Central Asian	0	1	>0.99
Male infants N (%)	3 (50)	5 (50)	>0.99
Gestational age at birth (days)	277 (272–283)	273 (265–274)	0.10
Exclusively breastfed N (%)	3 (50)	5 (50)	>0.99
Pre-pregnancy BMI (kg/m^2^)	23.0 ± 1.3	22.8 ± 3.2	0.91
Parity (previous pregnancies > 20 weeks) *	0.5 (0–1)	1 (1–3)	0.16
Antibiotic use 2 weeks pre-conception to 6 weeks post-partum N (%)	2 (33)	8 (80)	0.12
Probiotic use 2 weeks pre-concenption to 6 weeks post-partum N (%)	1 (17)	1 (10)	>0.99
Mastitis in the previous 6 weeks N (%)	1 (17)	0 (0)	0.38
Caesarean section N (%)	0 (0)	8 (80)	0.007
**Chronic Disease**			
Allergies (no anaphylaxis) N	1	4	0.59
Mental health disorder N	2	2	0.60
Endocrine disorder (inc. thyroid) N	1	2	>0.99
Asthma diagnosed ever N	0	4	0.23
Endometriosis N	0	2	0.50
Polycystic Ovarian Syndrome (PCOS) N	0	1	>0.99
Osteoarthritis N	0	1	>0.99
Dermatitis/eczema N	0	1	>0.99
Gilbert’s Syndrome N	0	1	>0.99
Gestational diabetes mellitus N	0	0	>0.99
Affected by >1 of the above N	1	5	0.31

**Table 2:** Comparison of library preparation and sequencing methods.

	Facility A	Facility B
Sequencing Platform	NovaSeq6000 (Illumina)	NovaSeq6000 (Illumina)
Sequencing length	2 × 150 bp	2 × 150 bp
Library preparation kit	Nextera DNA Flex Library Kit (Illumina #20018705)	Illumina DNA Prep (Illumina #20018705)
Library preparation platform	Mantis Liquid Handler (Formulatrix) and Epmotion (Eppendorf #507500301)	Zephyr NGS (Perkin Elmer)
Library quantification and quality control	Quanti-iT dsDNA HS Assay Kit (Invitrogen) and Agilent D1000 HS tapes (#5067-5582) on the TapeStation 4200 (Agilent # G2991AA)	Quant-iT ds DNA HS Assay kit (ThermoFisher Scientific) and QIAxcel Advanced System (#9002123) using QIAxcel DNA High Resolution Kit (#929002)
Library pooling	2nM per library	2nM per library
Library pool quantification	Qubit dsDNA HS assay kit (Invitrogen)	Qubit dsDNA HS assay kit (Invitrogen) on Qubit Flex Fluorometer
Library pool quality control	Agilent D1000 HS tapes (#5067-5582) on the TapeStation 4200 (Agilent #G2991AA)	QIAxcel Advanced System (#9002123) using QIAxcel DNA High Resolution Kit (#929002)

**Table 3:** Contents of broad-spectrum life-space probiotic (Melbourne, Australia).

Species	CFU/Capsule
*Lactobacillus rhamnosus* Lr-32	6 × 10^9^
*Lactobacillus rhamnosus* HN001	1 × 10^9^
*Lactobacillus plantarum* Lp-115	4.2 × 10^9^
*Lactobacillus rhamnosus* GG	4 × 10^9^
*Lactobacillus gasseri* Lg-36	5 × 10^8^
*Lactobacillus casei* Lc-11	3.2 × 10^9^
*Lactobacillus delbrueckii* subsp. *bulgaricus* Lb-87	2 × 10^8^
*Lactobacillus paracasei* Lpc-37	1.7 × 10^9^
*Lactobacillus reuteri* 1E1	2 × 10^8^
*Bifidobacterium animalis* subsp. *lactis* Bl-04	5 × 10^9^
*Bifidobacterium breve* Bb-18	8 × 10^8^
*Bifidobacterium longum* Bl-05	5 × 10^8^
*Bifidobacterium longum* subsp. *infantis* Bi-26	3 × 10^8^
*Bifidobacterium animalis* subsp. *lactis* HN019	1 × 10^9^
*Streptococcus thermophilus* St-21	3.4 × 10^9^

**Table 4:** Composition of the breastmilk microbiota and negative control as sequenced by Facility A and determined by MetaPhlAn3 [39]. Values represent percentage relative abundance.

Species	NegCon	BM A	BM B	BM C	BM D	BM E	BM F
*Cutibacterium acnes*	0	6.28	0	0	0	0	0
*Porphyromonas gingivalis*	2.06	0.70	0	2.04	2.09	0.74	1.08
*Gemella haemolysans*	0	0	0	0	0	3.89	0
*Staphylococcus aureus*	0	0	0	0	0	42.88	3.27
*Staphylococcus epidermidis*	0	2.81	4.85	1.02	1.87	2.50	3.18
*Enterococcus faecalis*	27.92	22.66	17.68	21.76	21.68	6.44	18.64
*Streptococcus agalactiae*	0	0	0	0	0	0.94	0
*Streptococcus mitis*	0	1.15	0	0	0	16.17	0
*Streptococcus mutans*	2.92	0.70	0	2.35	1.10	0.77	1.25
*Streptococcus oralis*	0	0	0	0	0	1.67	0
*Flavonifractor plautii*	0	0	0	0	0	0	10.10
*Veillonella atypica*	0	5.78	23.08	9.95	0	0	0
*Veillonella seminalis*	0	0	0	0	7.20	0	0
*Rhodobacter sphaeroides*	0	0.60	0	2.23	2.40	0	0.85
*Escherichia coli*	0.70	0	0	3.72	2.35	0	0.70
*Acinetobacter baumannii*	0	0	0	0	0.27	0	0
*Acinetobacter baylyi*	66.40	59.32	54.40	56.94	61.04	23.99	60.92
Total contaminants	100	83.38	72.08	86.81	88.26	31.95	82.60

Grey shading highlights bacteria identified in negative control.

**Table 5:** Composition of the breastmilk microbiota as sequenced by Facility B and determined by MetaPhlAn3 [39]. Four samples did not produce any identifiable bacterial DNA sequences. Values represent percentage relative abundance.

	BM G	BM H	BM I	BM J	BM K	BM L	BM M	BM N	BM O	BM P
*Bifidobacterium breve*	0	3.03	0	0	0	0	0	0	0	0
*Bifidobacterium longum*	0	2.03	0	0	0	0	0	0	0	0
*Corynebacterium kroppenstedtii*	0	0	100	0	0	0	0	0	0	0
*Rothia mucilaginosa*	0	3.46	0	0	0	0	0	0	0	0
*Cutibacterium acnes*	100	20.39	0	0	0	0	0	0	100	0
*Gemella haemolysans*	0	2.44	0	0	0	0	0	0	0	0
*Staphylococcus epidermidis*	0	5.34	0	0	100	0	0	0	0	0
*Staphylococcus hominis*	0	1.98	0	0	0	0	0	0	0	0
*Streptococcus mitis*	0	52.38	0	0	0	0	0	100	0	0
*Streptococcus oralis*	0	4.24	0	0	0	0	0	0	0	0
*Streptococcus parasanguinis*	0	2.37	0	0	0	0	0	0	0	0
*Streptococcus salivarius*	0	2.35	0	0	0	0	0	0	0	0

## Data Availability

The data presented in this study are available on request from the corresponding author.

## Supplementary Materials

The following supporting information can be downloaded at: https://www.mdpi.com/article/10.3390/nu15030696/s1, Figure S1: Gel image of 25 cycles of PCR for comparison of bacterial DNA extraction from three extraction kits and whole vs. skim milk. Figure S2: Comparison of Log(Bacterial/mL) of breastmilk samples at 6 weeks post-partum unaffected by antibiotics nAB (n = 6, vaginally delivered) and those affected by antibiotics in the previous 6 weeks (n =10, 8 Caesarean Delivered, 2 vaginally delivered).
