# Supplementary material for: Insufficient Evidence of a Breastmilk Microbiota at Six-Weeks Postpartum: A Pilot Study
Source: Nutrients. 2023 Jan 30;15(3):696. doi: 10.3390/nu15030696 (PMC9919471; doi:10.3390/nu15030696)
Supplement: Supplementary file 1 [file nutrients-15-00696-s001.zip › nutrients-2140287-supplementary.pdf]

Supplementary figures

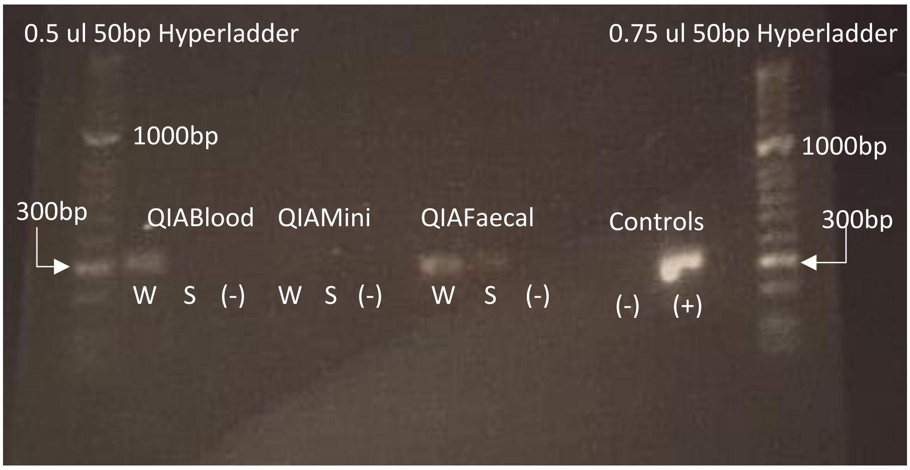

Figure S1: Gel electrophoresis of 25 cycles of PCR targeting a region of the 16s rRNA gene (expected length ~316bp) for comparison of bacterial DNA extraction. QIABlood = QIAamp DNA blood mini kit, QIAMini = QIAamp DNA mini kit, andQIAFaecal = QIAamp DNA PowerFaecal Pro DNA kit, Controls = PCR controls, W = whole milk, S = skim milk, (-) = negative control, (+) = positive control

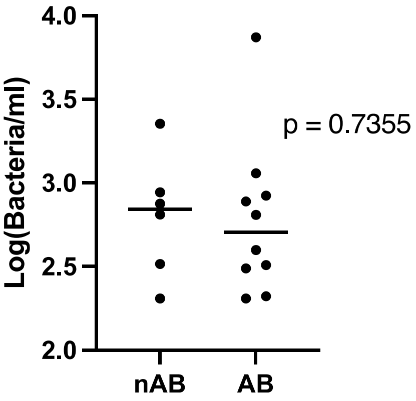

Figure S2: Comparison of Log(Bacterial/ml) of breastmilk samples at 6 weeks post-partum unaffected by antibiotics nAB (n = 6, vaginally delivered) and those affected by antibiotics in the previous 6 weeks (n =10, 8 Caesarean Delivered, 2 vaginally delivered)
